# Functional Metagenomics Reveals Gut Microbial Biosynthetic Potential for Essential Nutrients in Juvenile Chum Salmon

**DOI:** 10.1111/1462-2920.70448

**Published:** 2026-10-15

**Authors:** Subrata Kumar Ghosh, Mai A. Wassel, Marty Kwok‐Shing Wong, Susumu Hyodo, Koji Hamasaki

**Affiliations:** ^1^ Atmosphere and Ocean Research Institute The University of Tokyo Kashiwa Chiba Japan; ^2^ Department of Fishing and Post‐Harvest Technology, Faculty of Fisheries Chattogram Veterinary and Animal Sciences University Chattogram Bangladesh; ^3^ Genetics and Genetic Engineering Laboratory National Institute of Oceanography and Fisheries (NIOF) Cairo Egypt; ^4^ Collaborative Research Institute for Innovative Microbiology The University of Tokyo Bunkyo‐ku Tokyo Japan; ^5^ Department of Integrated Biosciences, Graduate School of Frontier Sciences The University of Tokyo Kashiwa Chiba Japan

**Keywords:** B‐vitamins, chum salmon, essential amino acids, functional potential, gut microbiome, KEGG module completion, shotgun metagenomics

## Abstract

The fish gut microbiota contributes to host nutrition, immunity and homeostasis, yet how its metabolic capacity relates to gaps in the host genome remains poorly characterized. Here we used shotgun metagenomic sequencing and KEGG module completion analysis to compare the biosynthetic potential of the gut microbiome with that of the host genome in juvenile chum salmon ( *Oncorhynchus keta* ) at 35 days posthatching, when exogenous feeding begins. Because small juvenile guts yield low microbial biomass and a high proportion of host DNA, we first optimized a protocol combining enzymatic lysis with NEBNext‐based host DNA depletion. This raised the bacterial sequence fraction from 11.3% to 41.2% and increased the number of KEGG Orthology identifiers mapped to modules from 1128 to 1532. Module Completion Ratio analysis revealed complementation in functional potential: the chum salmon genome was incomplete for *de novo* biosynthesis of essential amino acids (threonine, leucine, valine/isoleucine, lysine) and B‐vitamins (thiamine, riboflavin, biotin), whereas the gut metagenome encoded complete or near‐complete modules for these pathways. Sequences assigned to *Bartonella*‐like, *Acinetobacter* and *Enterococcus* lineages contributed most. These observations indicate that the gut microbiome encodes biosynthetic potential the host genome lacks, although demonstrating a nutritional benefit to the host will require experimental manipulation.

## Graphical abstract

At 35 days posthatching, the gut microbiome of juvenile chum salmon ( *Oncorhynchus keta* ) encodes complete biosynthetic pathways for essential amino acids (threonine, leucine, valine/isoleucine and lysine) and B‐vitamins (thiamine, riboflavin and biotin) that are absent or incomplete in the host genome. Bacterial taxa, including *Bartonella*, *Acinetobacter* and *Enterococcus,* are the main contributors to these functions, supporting host growth and survival during a critical early developmental window.

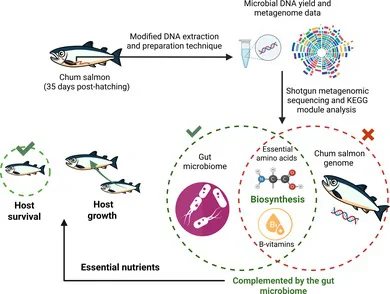

## 1 Introduction

The gastrointestinal tract of fish harbours a dense and diverse community of microorganisms that perform metabolic, structural and protective functions extending far beyond simple digestion (Egerton et al. 2018; Boutin et al. 2014). Besides supplying and transforming nutrients, gut bacteria contribute to the development of the intestinal epithelium, maturation of gut‐associated lymphoid tissue and innate immune signalling, competitive exclusion of pathogens, production of short‐chain fatty acids and other signalling metabolites, bile acid transformation and detoxification of dietary compounds (Nayak 2010; Rombout et al. 2011; Llewellyn et al. 2014; Luan et al. 2023). This breadth of influence has led to the gut microbiota being described as an ‘extra organ’ that supports metabolic homeostasis and overall health (Feng et al. 2018; Nayak 2010). Knowing not only which microbes are present but what they can actually do is therefore central to both basic fish biology and applied aquaculture.

Salmonids are well suited to this kind of functional investigation. Anadromy is the reason why. Pacific salmon spawn and hatch in fresh water but complete most of their somatic growth at sea, where productivity and prey availability are far higher than in natal streams. Moving between these two environments is not a passive drift. It requires smoltification, a coordinated remodelling of gill ionocytes, gut and kidney that switches the animal from ion uptake to ion excretion, and it carries a substantial energetic and physiological cost (McCormick 2013). Diet changes at the same time, from small freshwater invertebrates to marine zooplankton, and the gut microbiota turns over with it (Llewellyn et al. 2016; Dehler et al. 2017; Kale et al. 2025). Because the gut is simultaneously the organ of osmoregulatory transition, of nutrient acquisition and of microbial residence, the functional capacity of the gut microbiome during this window is of direct interest.

Chum salmon ( *Oncorhynchus keta* ) are an informative case within this group. They enter the sea earlier and at a smaller body size than most other Pacific salmon, so the fish that begin the marine phase are still developmentally immature and have little energetic reserve. Japanese fisheries depend heavily on hatchery release: about 262 hatcheries operate nationally, and releases peaked near two billion fry per year in 1983 before falling to roughly 1.3 billion in 2021, while adult returns fell from about 88 million in 1996 to 19 million in 2021 (Kitada and Kishino 2025). Understanding what limits juvenile quality before ocean entry is therefore not an abstract question. At the same time, salmonid genomes, like those of many other vertebrates, are incomplete for the *de novo* biosynthesis of some essential nutrients, including specific amino acids and B‐vitamins (Llewellyn et al. 2014; Olsvik et al. 2013). This implies that they rely on dietary intake and on microbial partners to fill these metabolic gaps.

We focused on 35 days posthatching for three connected reasons. First, this is when the yolk sac is exhausted and the fish switch to exogenous feeding, so the gut receives external substrate and an external microbial inoculum for the first time (Ghosh et al. 2025). Second, the gut epithelium and gut‐associated lymphoid tissue are still developing at this point, and early microbial colonization in salmonids is stage‐specific rather than a scaled‐down version of the adult community (Rombout et al. 1993, 2011; Lokesh et al. 2019; Llewellyn et al. 2016; Kale et al. 2025). Third, growth rate relative to body size is at its highest here, so per‐gram demand for essential amino acids and cofactors is correspondingly high. A microbial contribution to the essential nutrient pool, if it exists, should matter more at this stage than later in life, when the fish is larger, feeding on a richer marine diet and carrying a more established community. Adult microbiome data therefore cannot be used as a proxy for this window.

Most previous work on fish gut microbiota has focused on taxonomic profiling using 16S rRNA gene sequencing (Liu et al. 2016; Sullam et al. 2012). Although these studies have greatly advanced our understanding of which microbial lineages are present, they provide only indirect information about microbial functions. Functional potential can be inferred from 16S data using ancestral‐state tools such as PICRUSt2 (Douglas et al. 2020) or Tax4Fun2 (Wemheuer et al. 2020), and both are useful when only amplicon data exist. Their accuracy, however, depends on how well the sampled lineages are represented by sequenced reference genomes, which is a particular weakness for fish‐associated taxa. At the other end of the scale, metatranscriptomics and metaproteomics report on genes that are actually expressed, but they need higher‐quality and larger amounts of nucleic acid than a pooled juvenile gut readily provides and they cannot describe capacity that is present but not transcribed at the moment of sampling. We chose direct shotgun sequencing with KEGG module reconstruction because it measures gene content rather than predicting it, and because the KEGG module framework allows the host genome and the metagenome to be scored on exactly the same axis (Takami et al. 2012; Morita et al. 2007). That common axis is what makes a genome‐versus‐metagenome comparison interpretable.

Applying shotgun metagenomics to juvenile fish comes with practical challenges. Tiny gut sizes result in low total DNA yields, and a large proportion of that DNA is host‐derived. High host DNA content reduces the effective sequencing depth available for microbial genomes and distorts functional profiles. Conventional extraction methods that do not fully lyse bacteria, or that shear DNA, are also poorly suited to library preparation (Shi et al. 2022). This problem is well documented in salmonids specifically: recent work assembling a bacterial and viral genome catalogue from Atlantic salmon reported that host DNA dominates gut sequencing libraries and that specialized handling is needed to recover usable microbial signal from juvenile fish (Kale et al. 2025), and comparable difficulties have been reported across fish metagenomic studies more generally (Pereira‐Marques et al. 2019). Methylation‐based strategies for depleting host DNA have worked well in low‐biomass human and animal microbiome studies (Marotz et al. 2018), but they have not been thoroughly evaluated for small fish guts.

In this study, we addressed both the methodological and the biological problem. We first optimized and validated an enzymatic lysis and host DNA depletion protocol for whole guts of juvenile chum salmon. We then used the resulting metagenomic data together with the published chum salmon genome to estimate KEGG Module Completion Ratios for both the host and the microbiome and compared them directly to identify biosynthetic pathways that the host cannot complete but the microbiota can.

## 2 Materials and Methods

### 2.1 Ethics Statement

Chum salmon used in this study were provided by the Iwate Fisheries and Technology Center (Iwate Prefecture, Japan). Fish were euthanized in a buffered 0.1% (200 mg L^−1^) solution of tricaine methanesulfonate (MS‐222; Western Chemical Inc., Ferndale, WA, USA). All procedures followed the Guidelines for the Care and Use of Animals approved by the Animal Experiment Committee of The University of Tokyo and complied with the ARRIVE guidelines.

### 2.2 Fish Rearing and Sampling

Husbandry and sampling were carried out at the indoor aquarium facility of the Atmosphere and Ocean Research Institute (AORI), The University of Tokyo. Fertilized chum salmon eggs were obtained from a single artificial fertilization event at the Iwate Fishery Technology Center. Embryos were held in perforated plastic trays inside a 250 L rectangular recirculating tank supplied with continuously aerated fresh water (approximately 12°C, pH approximately 7.5, dissolved oxygen approximately 7 mg L^−1^). Temperature was maintained by an automatic heating and cooling system, and approximately 30% of the tank water was replaced daily.

After yolk absorption, fish were fed once daily with a commercial salmonid diet (KAIO3, Marubeni Nisshin Feed Co., Chita, Aichi, Japan; 51% crude protein, 8% crude fat, 3% crude fibre, 16% crude ash, 2% crude calcium, 1.5% crude phosphorus). Feeding was to apparent satiation, by which we mean that feed was delivered in small successive portions until the fish visibly stopped actively pursuing and ingesting pellets, after which no further feed was offered; uneaten pellets were siphoned out approximately 30 min after feeding to maintain water quality. This is a behavioural endpoint rather than a measured ration, so daily intake per fish was not quantified.

A single cohort from one fertilization event was reared in one tank, and 12 individuals (*n* = 12) were sampled at 35 days posthatching. Because the amount of gut tissue and microbial DNA obtainable from a single fry at this size is below the input requirement for shotgun library preparation, material from all 12 individuals was pooled into one homogenate, which was then split into four aliquots and taken through the four DNA pretreatment strategies described below. The four sequencing libraries are therefore four technical treatments of a single pooled biological sample, not four biological replicates, and this is the basis for the descriptive treatment of the comparisons in the Results. Sex was not recorded. Chum salmon at 35 days posthatching are sexually undifferentiated: gonadal differentiation in salmonids occurs considerably later in development, the gonads at this stage are morphologically indistinct and sex cannot be assigned visually or by dissection without molecular markers. Host sex was therefore neither a usable covariate nor a possible source of structured variation in this dataset.

Fish were euthanized with MS‐222 until opercular movement ceased (American Veterinary Medical Association 2007). The ventral surface was rinsed with 70% molecular‐grade ethanol, and a sterile midline incision was made from the pectoral fin to the cloacal pore. The entire intestinal tract, including gut contents, was aseptically removed, transferred to sterile tubes and stored at −80°C until DNA extraction.

One consequence of this design should be acknowledged at the outset. All fish received the same commercial formulated diet, and diet is among the strongest determinants of gut community composition and functional profile in salmonids (Gajardo et al. 2017; Wang et al. 2021). The community described here is therefore the community assembled under a specific, protein‐rich hatchery feed and it should be expected to differ from that of wild fry feeding on freshwater invertebrates, or of hatchery fry on an alternative formulation. Because the diet is constant across all four libraries, it cannot confound the methodological comparison, which is the primary purpose of the design. It does, however, bound the biological interpretation: the biosynthetic modules we recover reflect what this diet‐associated community encodes, and whether the same modules would be recovered on a different feed is an open and directly testable question.

### 2.3 DNA Extraction and Optimization for Shotgun Sequencing

DNA was extracted from the pooled gut homogenate using the Isoil DNA extraction kit (Nippon Gene Co. Ltd., Japan), with modifications to improve cell lysis while limiting DNA shearing. Each sample was homogenized in 450 μL Buffer HE and 450 μL enzymatic lysis buffer containing 50 μL lysozyme (20 mg mL^−1^) and incubated at 37°C for 30 min to lyse bacterial cell walls, including those of Gram‐positive bacteria. Proteinase K (50 μL, 20 mg mL^−1^) was added, briefly vortexed and incubated at 55°C for 30 min. The mixture was centrifuged at 12,000*g* for 15 min and the supernatant processed following the manufacturer's protocol. Mechanical disruption was avoided throughout to minimize fragmentation. DNA concentration and purity were measured on a Qubit Spectrophotometer and NanoDrop spectrophotometer, respectively, before and after each pretreatment (Table S1). The untreated extract yielded 650.8 ng μL^−1^ with an A260/A280 ratio of 1.92 and an A260/A230 ratio of 2.34.

### 2.4 Host DNA Depletion and Comparison of Pretreatment Strategies

Four pretreatment strategies were applied. (i) Original DNA: used directly for library preparation without enrichment or amplification. (ii) NEBNext enrichment: DNA was treated with the NEBNext Microbiome DNA Enrichment Kit (E2612L; New England Biolabs Inc., USA) according to the manufacturer's instructions. The kit exploits methylation differences between eukaryotic and prokaryotic DNA, using an MBD2‐Fc fusion protein to bind methylated host DNA, which is removed with magnetic beads (Marotz et al. 2018). (iii) NEBNext enrichment followed by 17‐cycle PCR: enriched DNA was fragmented (Covaris shearing, peak size approximately 350 bp), ligated to adapters and amplified for 17 cycles using the MGIEasy Universal DNA Library Prep Set. (iv) NEBNext enrichment followed by whole‐genome amplification (WGA): enriched DNA was amplified with the GenomiPhi V2 kit (25,660,022; Cytiva, Marlborough, USA) by multiple‐displacement amplification (Lasken 2012). Library preparation followed the same workflow after fragmentation for all treatments, and size distributions (approximately 200–700 bp) were confirmed before sequencing. Libraries were sequenced on an MGI DNBSEQ platform.

Host DNA depletion carries a substantial cost in total DNA mass, and we record it here because it constrains how the protocol can be used. Depletion reduced the concentration of the extract from 650.8 to 28.1 ng μL^−1^, a 23‐fold decrease, so roughly 4% of the starting DNA mass survived the procedure. Some of this loss is the intended removal of host DNA, which made up 88.7% of annotated sequences before treatment (Figure 3), but not all of it: if the sequence‐level composition is taken as a rough proxy for mass composition, complete retention of the microbial fraction would have left about 11% of the starting mass rather than 4%, which implies that a little over a third of the microbial DNA was recovered. Bead‐based capture is not perfectly selective, and unmethylated DNA is carried down with the beads along with the methylated host fraction. The A260/A230 ratio also fell from 2.34 to 1.27 after depletion, consistent with salt carried over from the binding and wash steps, although this did not prevent successful library construction. WGA restored mass to 243.3 ng μL^−1^ and the A260/A230 ratio to 2.10, but at the cost of sequence yield and functional recovery reported below. The NEBNext + PCR library was amplified during library preparation, downstream of the depleted eluate, so it has no separate posttreatment quantification. All values are given in Table S1.

### 2.5 Metagenome Processing, Annotation and Functional Module Analysis

Quality‐filtered reads were processed through the Genomaple Submission Data Maker (GSDM), which performs trimming, paired‐end assembly, gene finding, translation to amino acid sequences and random sampling, and outputs a multi‐FASTA file. These datasets were analysed with the Genome Metabolic and Physiological Potential Evaluator (GenomapleTM, formerly MAPLE) version 2.3.2 (Takami et al. 2012, 2016; Arai et al. 2018; Okubo et al. 2021), which assigns genes to KEGG Orthology (KO) identifiers, maps KOs to modules and calculates the Module Completion Ratio.

The analytical strategy here is gene‐centric rather than genome‐centric, and it is worth being explicit about what that means for the summary statistics that can be reported. GSDM merges overlapping read pairs and predicts genes on the resulting sequences; it does not perform *de novo* contig assembly with an assembler such as metaSPAdes or MEGAHIT, and no differential‐coverage binning was carried out. Consequently, there are no contigs in the conventional sense, no N50 or contig length distribution and no metagenome‐assembled genomes, so completeness and contamination statistics of the kind produced by CheckM cannot be reported for this dataset. The appropriate quality metrics for a gene‐centric pipeline are the number of query amino acid sequences, the proportion of those sequences receiving a KO assignment and the number of KOs successfully mapped to KEGG modules, together with the rarefaction behaviour of these counts. All of these are reported in Figure 2 and compiled in Table S1.

We chose this approach deliberately given the sample. Even after depletion, close to 60% of annotated sequences remained eukaryotic (Figure 3), and the library derives from a pooled, unreplicated sample. Assembly and binning under these conditions would be expected to yield few bins, of low and uneven completeness, with a substantial risk of chimeric bins formed from co‐abundant relatives; differential‐coverage binning in particular is not applicable without multiple samples. A gene‐centric approach avoids making genome‐level claims that the data cannot support, and the KEGG module framework it feeds into is what allows the host genome and the metagenome to be scored on one common axis. We regard assembly and binning as the natural next step once the host fraction can be pushed lower, and Table S1 is provided so that the present dataset can be benchmarked against future assembly‐based work on the same species.

### 2.6 Calculation of Module Completion Ratio

For each KEGG module the MCR was defined as the percentage of required KOs present in the dataset. An MCR of 100% indicates that all KOs required by the Boolean logic of the module are represented and the pathway is considered complete. MCR values were calculated in GenomapleTM according to the Boolean algebra‐like equations of Takami et al. (2012), with minor adjustments for recent KEGG updates.

### 2.7 Taxonomic Profiling

Taxonomic composition was estimated from ribosomal proteins annotated within Genomaple using the KEGG Automatic Annotation Server module against its reference genome set. Ribosomal proteins were used because they are near‐universal, present in low and predictable copy number and strongly conserved, which makes them a stable basis for relative abundance estimation from unassembled sequence data; equivalent single‐copy marker‐gene strategies underlie widely used profilers. Relative abundances of bacterial and archaeal taxa were calculated per treatment from these annotations, with host sequences excluded, so reported values are proportions within the prokaryotic fraction.

Classification against the Genome Taxonomy Database using GTDB‐Tk (Chaumeil et al. 2020; Parks et al. 2022) would be preferable and is now standard practice, and we considered it. GTDB‐Tk classifies genomes or metagenome‐assembled genomes rather than unassembled reads or predicted genes, and requires an input bin of sufficient completeness to place on the reference tree. Because no bins were produced for the reasons set out above, GTDB‐Tk could not be applied to this dataset. Related limitations follow: average nucleotide identity, the accepted criterion for prokaryotic species boundaries (Konstantinidis and Tiedje 2005; Jain et al. 2018), cannot be computed without genomes, so no species‐level assignment reported here is ANI‐validated. Taxon names in the Results and Discussion accordingly denote the closest reference genome in the annotation database, and are qualified as such throughout. Applying GTDB‐Tk and ANI to binned genomes from an improved, replicated dataset is a defined objective of ongoing work.

### 2.8 Domain‐Level Partitioning of Sequences

To quantify the effect of host depletion on library composition, annotated sequences from each of the four libraries were partitioned by domain of origin using the same Genomaple annotation output, and the percentages of bacterial and eukaryotic sequences were calculated for each library (Figure 3). Eukaryotic sequences in these samples derive overwhelmingly from the host. This partition is reported separately from the community composition analysis described above, in which host sequences are excluded.

### 2.9 Comparative Functionome Analysis

To examine metabolic complementation between host and microbiome, the functionomes of the chum salmon genome and the gut metagenome were compared on the basis of KEGG modules and their MCR values. The NEBNext + 17‐cycle PCR dataset was used because it yielded more than one million amino acid sequences and the highest number of KOs mapped to modules (1532). Chum salmon genome data (117,599 protein‐coding sequences, 100% ribosome completeness) were retrieved from NCBI. Both datasets were processed through GenomapleTM for KO assignment and MCR calculation, and comparative histograms were generated with the GenomapleTM Graph Maker tool. Module Completion Ratios for every KEGG module are provided in full for the chum salmon genome (Table S3), the original DNA library (Table S4), the NEBNext + 17‐cycle PCR library (Table S5) and the NEBNext + WGA library (Table S6).

## 3 Results

### 3.1 Sequencing Results and Comparison of Pretreatment Methods

We compared four DNA pretreatment strategies: (i) original DNA with no host depletion, (ii) NEBNext host DNA depletion, (iii) NEBNext depletion followed by 17‐cycle PCR amplification and (iv) NEBNext depletion followed by WGA of the bacterial fraction. The bioinformatic workflow used to process the resulting reads is shown in Figure 1.

**FIGURE 1:**
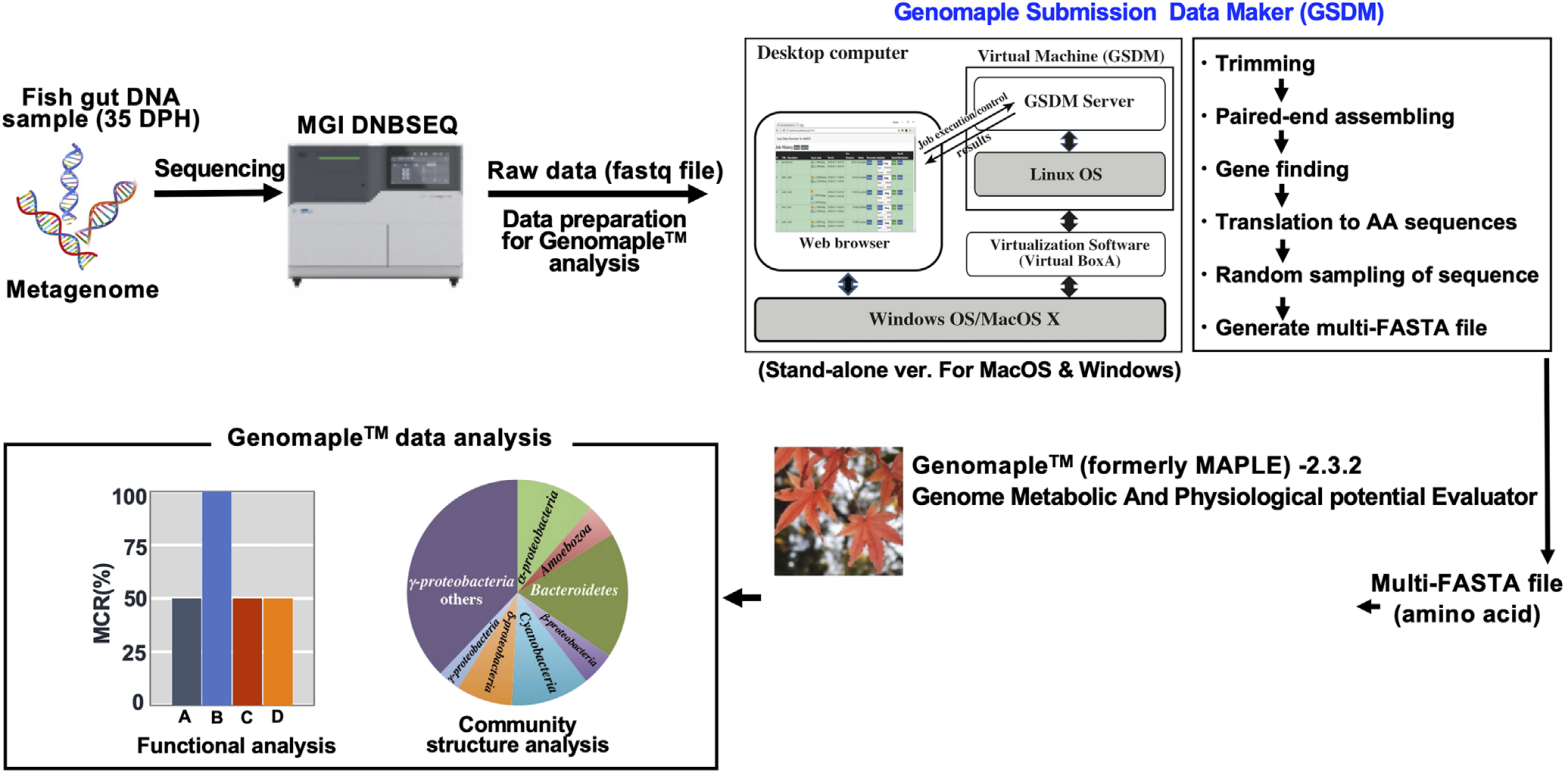
Overview of the bioinformatic pipeline used to characterize the juvenile chum salmon gut functionome. Total DNA extracted from pooled whole gut samples of juvenile chum salmon ( *Oncorhynchus keta* ) at 35 days posthatching was sequenced on an MGI DNBSEQ platform. Raw fastq files were processed through the Genomaple Submission Data Maker (GSDM), which performs trimming, paired‐end assembly, gene finding, translation to amino acid sequences and random sampling, and outputs a multi‐FASTA file of amino acid sequences. These sequences were submitted to Genomaple version 2.3.2 for functional analysis based on Module Completion Ratios (MCR, %) and for microbial community structure profiling based on conserved marker genes.

Rarefaction curves showed that the number of assigned KO identifiers and the number of KOs mapped to functional modules differed markedly among treatments (Figure 2A–D). The two strategies that included host DNA depletion, NEBNext alone (Figure 2B) and NEBNext plus 17‐cycle PCR (Figure 2C), both produced approximately one million translated amino acid sequences (998,957 and 999,458, respectively). The highest number of KOs mapped to KEGG modules (1532) was obtained from the NEBNext + PCR treatment, closely followed by the NEBNext‐only treatment (1499). The untreated original DNA sample yielded 1128 KOs in modules from 653,543 query sequences (Figure 2A), and the WGA sample only 666 KOs from 338,389 sequences (Figure 2D). Summary statistics for all four libraries, including query sequence counts, KOs assigned to genes, KOs mapped to modules and the corresponding domain‐level sequence composition, are compiled in Table S1.

**FIGURE 2:**
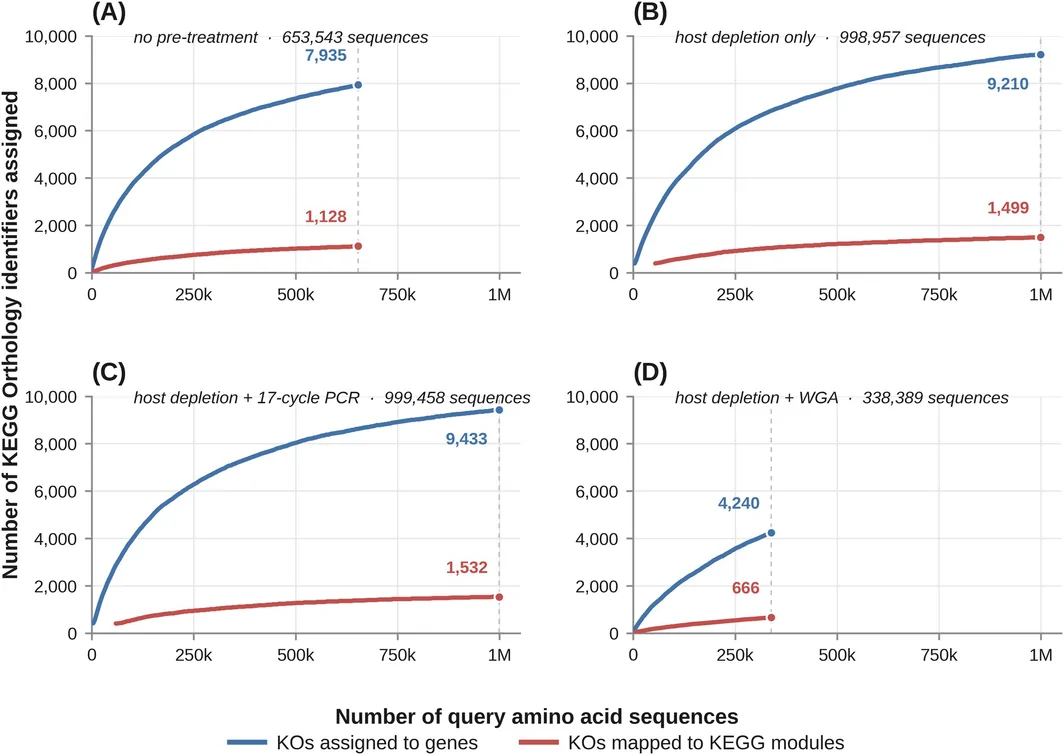
Effects of DNA pretreatment on metagenomic depth and functional annotation in the juvenile chum salmon gut. Rarefaction curves showing the number of KEGG Orthology (KO) identifiers assigned to genes (blue lines) and the number of KOs mapped to KEGG modules (red lines) as a function of the cumulative number of query amino acid sequences. All four panels share the same axes so that the libraries can be compared directly; the dashed vertical line in each panel marks the point at which sequencing of that library ended, and final values are given beside each endpoint. (A) Original DNA, no postextraction treatment (653,543 sequences; 7935 KOs in genes; 1128 KOs in modules). (B) NEBNext host DNA depletion (998,957 sequences; 9210 KOs in genes; 1499 KOs in modules). (C) NEBNext depletion followed by 17‐cycle PCR amplification (999,458 sequences; 9433 KOs in genes; 1532 KOs in modules). (D) NEBNext depletion followed by whole‐genome amplification of the bacterial fraction using the GenomiPhi kit (338,389 sequences; 4240 KOs in genes; 666 KOs in modules).

### 3.2 Host DNA Depletion Raises the Bacterial Sequence Fraction Almost Fourfold

We partitioned annotated sequences from each library by domain of origin using the same Genomaple annotation (Figure 3). In the untreated library, only 11.3% of annotated sequences were bacterial and 88.7% were eukaryotic, confirming that host DNA overwhelmingly dominates a juvenile chum salmon gut extract. NEBNext depletion raised the bacterial fraction to 41.2%, a 3.6‐fold enrichment. The two amplified libraries fell between these values, at 35.4% bacteria for NEBNext + PCR and 35.7% for the WGA library, indicating that neither amplification step recovered additional host sequence nor improved on depletion alone. The residual eukaryotic fraction after depletion, still close to 60%, shows that methylation‐based depletion reduces host DNA substantially without eliminating it, which is consistent with reports from other low‐biomass systems (Marotz et al. 2018; Pereira‐Marques et al. 2019). This residual host signal is the main reason we did not attempt genome binning (see Experimental Procedures). The gain in microbial signal comes at a cost in total DNA: depletion reduced the extract from 650.8 to 28.1 ng μL^−1^, so about 4% of the starting mass remained (Table S1). For a pooled gut sample, this was still enough for library preparation, but it sets a practical floor on how little starting material the protocol can tolerate, and it is the reason we tested the two amplification routes.

**FIGURE 3:**
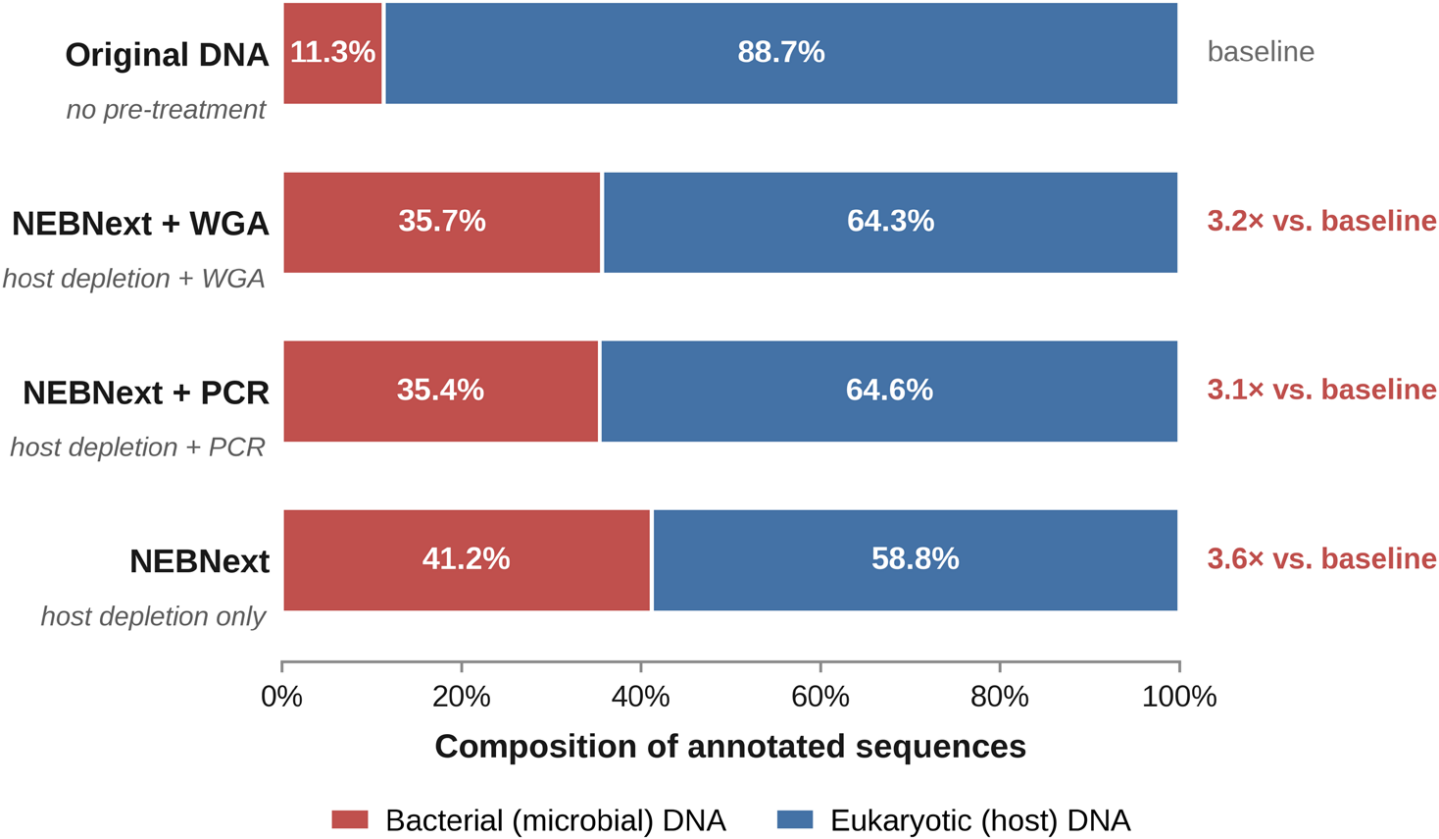
Host DNA depletion shifts the composition of the sequencing libraries toward the microbial fraction. Each horizontal bar represents one library prepared from the same pooled juvenile chum salmon gut extract at 35 days posthatching and taken through a different pretreatment. Bars are split into the percentage of annotated sequences of bacterial (microbial) origin, shown in red on the left and the percentage of eukaryotic origin, shown in blue on the right; the eukaryotic sequences derive almost entirely from the host. Because the two categories sum to 100% in every library, the position of the boundary shows the composition of each library directly. Without pretreatment, only 11.3% of annotated sequences were bacterial. Methylation‐based host DNA depletion with the NEBNext Microbiome DNA Enrichment Kit raised the bacterial fraction to 41.2%, and the two amplified libraries (NEBNext followed by PCR or by whole‐genome amplification) reached 35.4% and 35.7%, respectively. The fold change of the bacterial fraction relative to the untreated library is given to the right of each bar. Values are percentages of total annotated sequences per library.

### 3.3 Microbial Community Structure of the Chum Salmon Gut Microbiome

To evaluate whether host DNA depletion and amplification altered community composition, we compared microbial profiles across the four treatments using conserved ribosomal proteins (Figure 4A). These compositional analyses were computed on bacterial and archaeal marker genes only; host sequences were excluded before relative abundances were calculated, so the values in Figure 4 are proportions within the prokaryotic fraction and are not directly diluted by host DNA. What depletion changes is the effective sequencing depth available to that prokaryotic fraction. With only 11.3% of sequences bacterial in the untreated library, marker genes from lower‐abundance taxa are sampled sparsely or missed altogether, and the resulting profile is dominated by whichever few taxa are abundant enough to survive that sampling. Raising the bacterial fraction to 41.2% increases the number of marker genes recovered per taxon and therefore stabilizes the estimate, which is what we observe.

**FIGURE 4:**
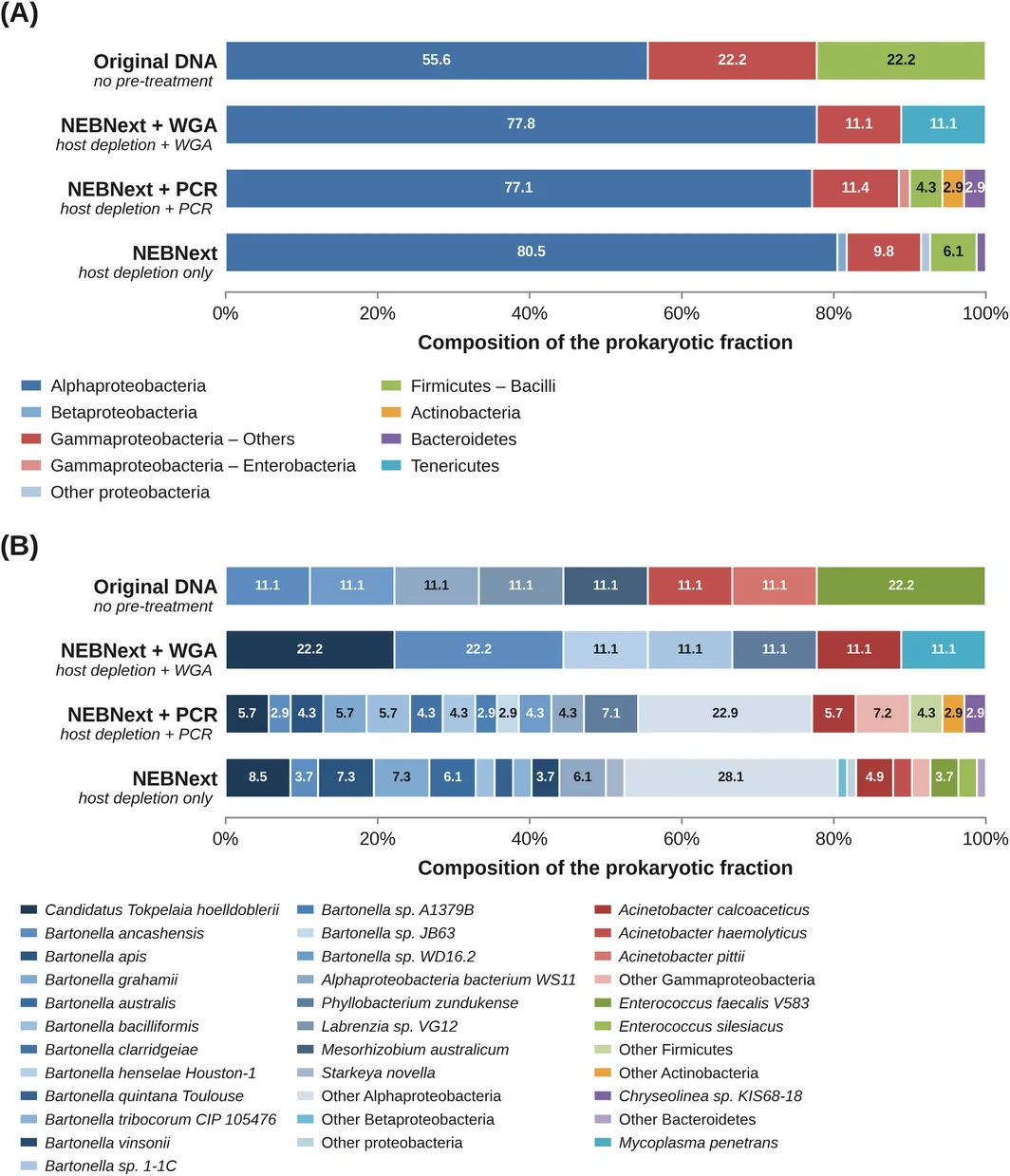
Taxonomic composition of the juvenile chum salmon gut microbiome under four DNA pretreatments. Community profiles were reconstructed from ribosomal protein assignments in four metagenomic libraries prepared from a single pooled gut homogenate of 12 fish sampled at 35 days posthatching. (A) Composition at phylum level, with Proteobacteria resolved to class. (B) Composition at species level. Every taxon that the annotation resolved to species is shown individually; sequences that could not be resolved beyond class or phylum are grouped as ‘Other’ within their own lineage rather than pooled into a single category, so the unresolved fraction can still be read taxonomically. Colours group taxa by lineage: Blue tones for Alphaproteobacteria, red for Gammaproteobacteria, green for Firmicutes, purple for Bacteroidetes, orange for Actinobacteria and teal for Tenericutes. Host sequences were removed before these values were calculated. Taxon names denote the closest reference genome in the Genomaple database rather than average nucleotide identity‐validated species assignments, and proportions of low‐abundance taxa vary between treatments and should be interpreted qualitatively.

At the class level, Alphaproteobacteria and Gammaproteobacteria were consistently dominant. Alphaproteobacteria comprised 55.6% of the original DNA library and 80.5% and 77.1% in the NEBNext‐treated and NEBNext + PCR libraries, respectively. Tenericutes, the phylum containing *Mycoplasma*, were detected in the original DNA library at modest relative abundance and were lower in the depleted libraries. At the species level (Figure 4B), this signal corresponded to a lineage whose closest reference genome in the annotation database was *Mycoplasma penetrans* . We note explicitly that this is a best‐match assignment from homology search and not a validated species‐level identification; without genome bins and average nucleotide identity values, which are the accepted basis for prokaryotic species delineation (Konstantinidis and Tiedje 2005; Jain et al. 2018), we can state only that a *Mycoplasma*‐affiliated lineage was present. The same caution applies to every species‐level name reported below, and we have used ‘‐like’ or ‘affiliated with’ phrasing accordingly.

The NEBNext‐treated and NEBNext + PCR libraries yielded more diverse and internally consistent profiles than the untreated and WGA libraries. Taxa such as *Acinetobacter calcoaceticus* ‐like sequences, *Candidatus* Tokpelaia hoelldoblerii and *Bartonella ancashensis*‐like sequences, detected at low or zero relative abundance in the untreated sample, were recovered and quantified in the enriched libraries. Figure 4 does show treatment‐dependent variation, particularly among low‐abundance taxa and in the WGA library, where multiple‐displacement amplification is known to amplify templates unevenly. What we can support is a narrower statement: the identity and rank order of the dominant taxa were preserved across the two depleted libraries, whereas rare‐taxon estimates were not stable and should not be interpreted quantitatively. Bias affecting rare taxa can be mitigated by sequencing biological replicates rather than pooled samples, by including a defined mock community as an internal control through the full depletion and library workflow, and by treating amplification‐based libraries as qualitative for anything below a few percent relative abundance. We adopt the third of these here and recommend the first two for future work. On the basis of functional depth, the NEBNext + PCR library was selected for the metabolic analyses that follow. The full species‐level composition of each library is given in Table S2.

### 3.4 Comparative Functionome Analysis of Chum Salmon Genome and Gut Microbiome

To explore how the metabolic potential of the microbiome relates to that of the host, we compared MCRs between the chum salmon genome and the gut metagenome (Figure 5A,B). Many central metabolic pathways, including glycolysis, the tricarboxylic acid cycle and core nucleotide metabolism, were well represented in both. In contrast, MCRs for several biosynthetic pathways for essential amino acids (Figure 5A) and B‐vitamins (Figure 5B) were low or absent in the host genome but high in the gut metagenome. Beyond the pathways examined in detail below, the comparison also identified cysteine biosynthesis and the plant/bacterial‐type haem biosynthesis pathway, the latter coexisting with the host's own animal/fungal‐type haem pathway. Module names highlighted in blue in Figure 5A,B mark all such cases. The complete set of Module Completion Ratios underlying this comparison is given for the chum salmon genome in Table S3 and for the NEBNext + PCR library in Table S5.

**FIGURE 5:**
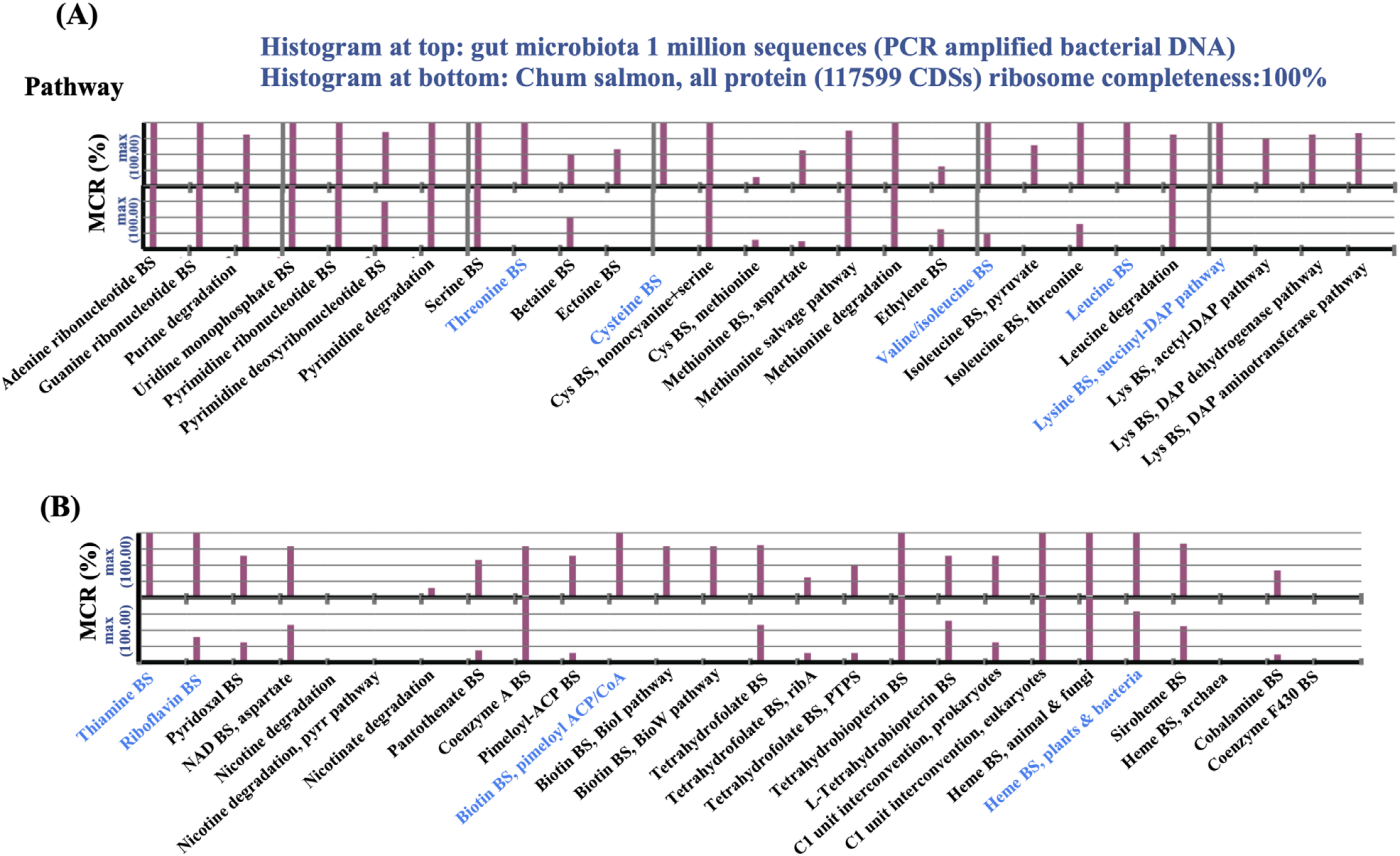
Comparative module completion patterns for chum salmon and its gut microbiome. Histograms showing the Module Completion Ratio of KEGG modules reconstructed from the juvenile chum salmon gut metagenome (NEBNext + 17‐cycle PCR treatment; top panels) and from the chum salmon host genome (117,599 protein‐coding sequences, 100% ribosome completeness; bottom panels), grouped by functional category. (A) Amino acid biosynthesis pathways. (B) Cofactor and vitamin biosynthesis pathways. Module names highlighted in blue indicate pathways that are incomplete or absent in the host genome but fully or nearly complete in the gut metagenome.

### 3.5 Biosynthesis of Essential Amino Acids

We next examined KEGG modules for essential amino acid biosynthesis, recording both the MCR and the reference genomes to which contributing sequences were assigned (Figure 6).

**FIGURE 6:**
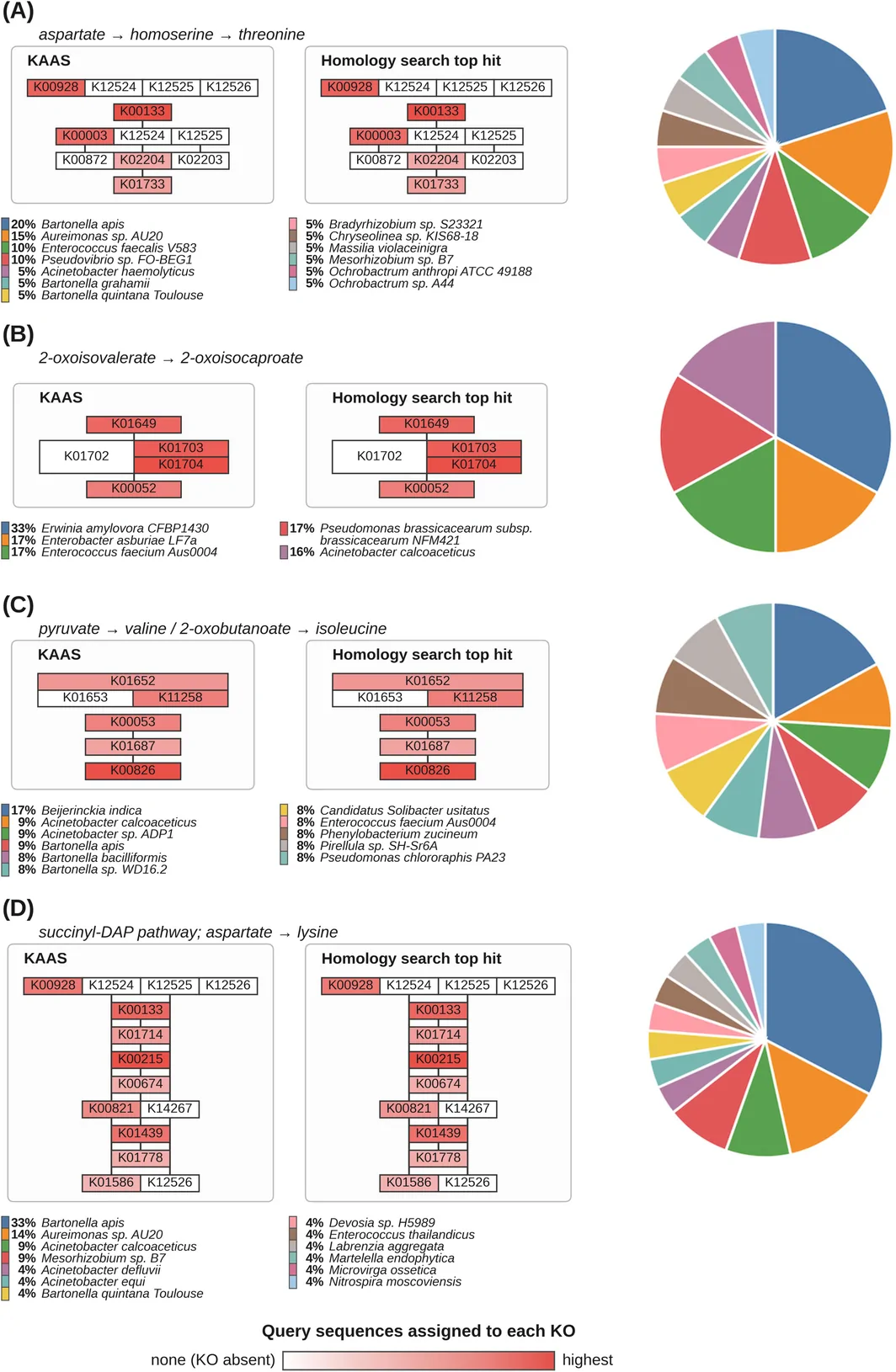
Bacterial taxa contributing to essential amino acid biosynthesis modules in the gut microbiome of juvenile chum salmon. Reconstruction of four KEGG modules for essential amino acid biosynthesis from the 35 DPH chum salmon gut metagenome (NEBNext + 17‐cycle PCR library). (A) Module M00018, threonine biosynthesis from aspartate via homoserine. (B) Module M00432, leucine biosynthesis from 2‐oxoisovalerate. (C) Module M00019, valine/isoleucine biosynthesis from pyruvate. (D) Module M00016, lysine biosynthesis via the succinyl‐DAP pathway. For each module, the left‐hand diagram shows the reaction network as annotated by the KEGG Automatic Annotation Server (KAAS) and the right‐hand diagram the same network annotated by the homology‐search top hit. Each box represents one KEGG Orthology (KO) identifier required by the module, and boxes drawn side by side within a row are alternative KOs that satisfy the same step. Box fill indicates the number of query sequences assigned to that KO, from white (KO not detected in the dataset) to saturated red (highest number of assigned sequences), as shown by the scale at the foot of the figure. Pie charts show the proportion of module completion attributable to each reference genome, weighted by Module Completion Ratio; the colour key beneath each panel gives the corresponding percentages. Taxon names denote the closest available reference genome in the Genomaple annotation database and are not average nucleotide identity‐validated species assignments.

Module M00018 (threonine biosynthesis from aspartate via homoserine) was incomplete in the chum salmon genome but complete in the gut metagenome. Contributions came mainly from sequences assigned to *Bartonella apis* (20%), *Aureimonas* sp. AU20 (15%), *Enterococcus faecalis* V583 (10%) and *Pseudovibrio* sp. FO‐BEG1 (10%) (Figure 6A). Module M00432 (leucine biosynthesis from 2‐oxoisovalerate) followed a similar pattern, with *Erwinia amylovora* CFBP1430 (33%), *Enterococcus faecium* Aus0004 (17%), *Enterobacter asburiae* LF7a (17%), *Pseudomonas brassicacearum* subsp. *brassicacearum* NFM421 (17%) and *A. calcoaceticus* (16%) (Figure 6B). Module M00019 (valine/isoleucine biosynthesis from pyruvate) was incomplete in the host genome but highly complete in the metagenome, with contributions from *Beijerinckia indica* (17%), *B. apis* (9%), *A. calcoaceticus* (9%), *Bartonella bacilliformis* (8%) and *Bartonella* sp. WD16.2 (8%) (Figure 6C). Module M00016 (lysine biosynthesis via the succinyl‐DAP pathway) was similarly dominated by microbial genes, with *B. apis* (33%), *Aureimonas* sp. AU20 (14%), *A. calcoaceticus* (9%) and *Mesorhizobium* sp. B7 (9%) (Figure 6D). As above, these names identify the closest available reference genome for each contributing sequence rather than a confirmed species identification.

### 3.6 Biosynthesis of Essential Vitamins

We also examined modules for thiamine (B1), riboflavin (B2) and biotin (B7) biosynthesis (Figure 7). Module M00127 (thiamine biosynthesis) was largely completed by bacterial genes, with contributions from *A. calcoaceticus* (combined slices, approximately 28%), *Bartonella* sp. JB63 (combined, 14%), *B. bacilliformis* (8%), *Pelagibaca abyssi* (8%) and *Bartonella* sp. WD16.2 (7%) (Figure 7A). Module M00125 (riboflavin biosynthesis from GTP) received contributions from *A. calcoaceticus* (11%), *Bartonella australis* (11%), *Bartonella tribocorum* CIP 105476 (11%) and *Mesorhizobium* sp. B7 (11%) (Figure 7B). For biotin, module M00123 had a higher MCR in the microbiome than in the host genome, but the dominant assignments were to intracellular bacterial endosymbionts rather than free‐living gut bacteria: *Wolbachia* strain wCle, an endosymbiont of the bed bug *Cimex lectularius* (25%), *Candidatus* Fokinia solitaria (19%) and the *Cardinium* endosymbiont cEper1 of the parasitoid wasp *Encarsia pergandiella* (13%) (Figure 7C). These assignments are discussed critically below. Overall, the vitamin modules mirror the amino acid result: the chum salmon genome lacks complete pathways for these B‐vitamins while the gut metagenome encodes the genes needed to complete them.

**FIGURE 7:**
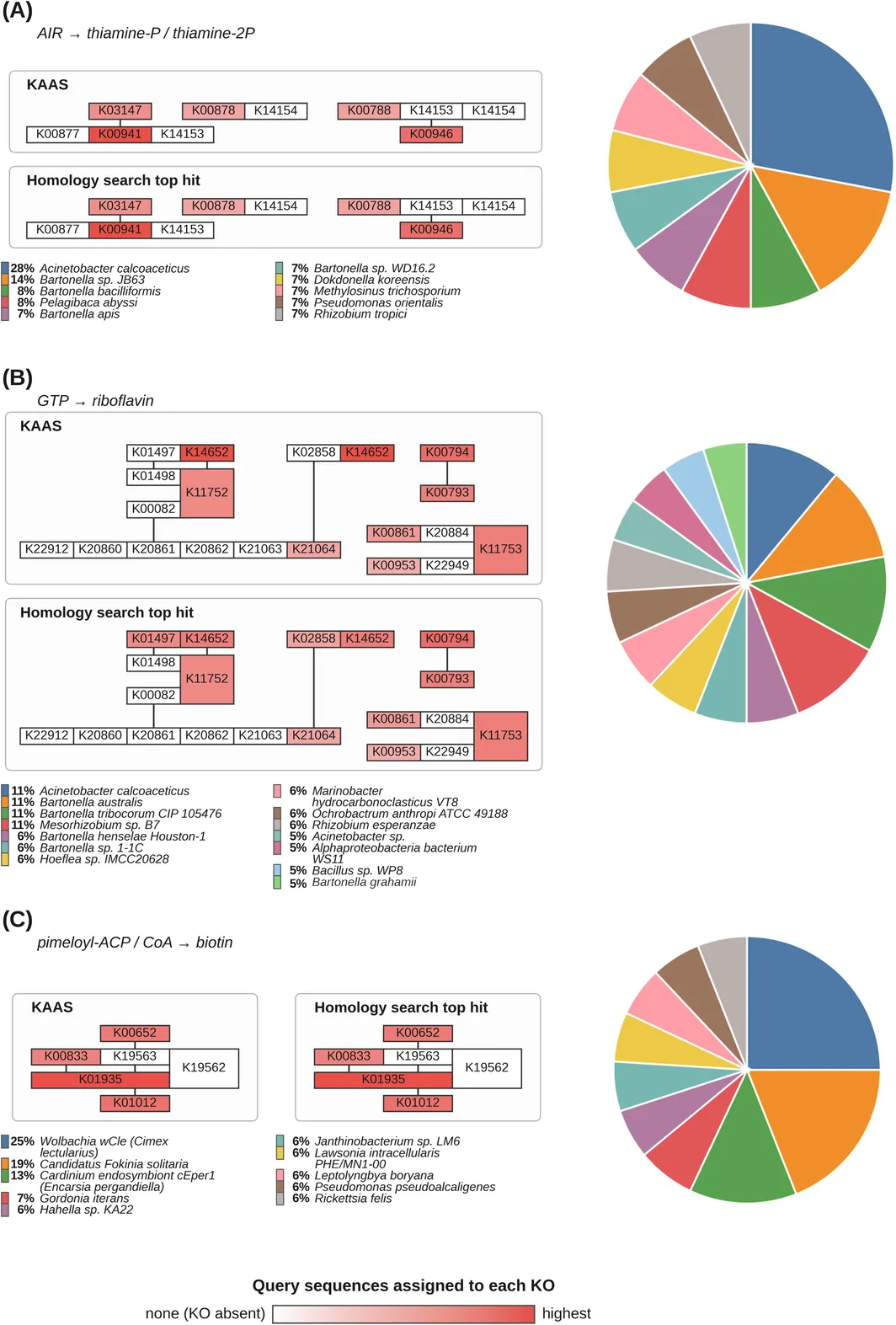
Bacterial taxa contributing to thiamine, riboflavin and biotin biosynthesis modules in the gut microbiome of juvenile chum salmon. Reconstruction of three KEGG modules for B‐vitamin biosynthesis from the 35 DPH chum salmon gut metagenome (NEBNext + 17‐cycle PCR library). (A) Module M00127, thiamine (vitamin B1) biosynthesis from AIR to thiamine‐P/thiamine‐2P. (B) Module M00125, riboflavin (vitamin B2) biosynthesis from GTP. (C) Module M00123, biotin (vitamin B7) biosynthesis from pimeloyl‐ACP/CoA. Diagrams, box fills and the colour scale are as described for Figure 6; for modules A and B the KAAS and homology‐search reconstructions are stacked vertically to accommodate the width of the network. Pie charts show the proportion of module completion attributable to each reference genome, weighted by Module Completion Ratio, with percentages given in the colour key beneath each panel. Taxon names denote the closest available reference genome rather than a confirmed species identification. Box fill indicates the number of query sequences assigned to that KO, from white (KO not detected in the dataset) to saturated red (highest number of assigned sequences), as shown by the scale at the foot of the figure.

## 4 Discussion

### 4.1 Methodological Advancement for Low‐Biomass Metagenomics

Applying shotgun metagenomics to very small fish is difficult because gut tissue is tiny, microbial biomass is low and host DNA dominates libraries. Among the methods tested, gentle enzymatic lysis combined with methylation‐based host DNA depletion produced approximately one million translated amino acid sequences and the highest number of KOs mapped to modules (1532). The domain‐level partition in Figure 3 quantifies why this matters: depletion moved the bacterial sequence fraction from 11.3% to 41.2%, so roughly four times as much of the sequencing effort was spent on the organisms of interest. Neither PCR nor WGA improved on depletion alone in this respect, and WGA in particular reduced both sequence yield and functional recovery, which is consistent with the uneven template amplification characteristic of multiple‐displacement approaches (Lasken 2012). Similar methylation‐based depletion strategies have been recommended for low‐biomass samples in other systems (Marotz et al. 2018; Pereira‐Marques et al. 2019), and our results extend that recommendation to juvenile fish guts. The residual eukaryotic fraction of roughly 60% after depletion is worth stating plainly because it sets the ceiling on what this workflow can currently achieve and defines the target for further method development in salmonids (Kale et al. 2025). The second constraint is mass. Depletion left roughly 4% of the starting DNA (Table S1), which a pooled sample can absorb but a single juvenile gut probably cannot, so applying this workflow to individual fry will require either more sensitive library chemistry or a depletion step with better microbial recovery.

### 4.2 Complementation in Functional Potential and the Generality of the Finding

The central result is a quantitative complementation in functional potential between the chum salmon genome and its gut microbiome. Comparing MCRs on the same KEGG module scale shows that the fish genome is incomplete for several biosynthetic pathways while the gut metagenome carries complete versions of the same modules, specifically for threonine, leucine, valine/isoleucine, lysine, thiamine, riboflavin and biotin. This is consistent with the broader understanding that vertebrate genomes, salmonids included, have lost the capacity to synthesize many essential amino acids and B‐vitamins and depend on diet and on symbiotic microbes (Llewellyn et al. 2014; Olsvik et al. 2013; Wang et al. 2021).

We want to be precise about what this does and does not show. A complete KEGG module means the genetic capacity is present in the community. It does not establish that the pathway is transcribed, that the product is released, that the host absorbs it, or that the host would grow more slowly without it. Establishing those links requires manipulation rather than observation. Gnotobiotic zebrafish work provides the template: germ‐free animals show altered nutrient absorption and metabolic gene expression that is restored on conventionalization (Rawls et al. 2004), and microbiota have been shown to regulate intestinal fatty acid absorption directly (Semova et al. 2012). No equivalent germ‐free system exists for chum salmon, so for the present we describe biosynthetic potential and treat the nutritional consequence as a hypothesis rather than a result. In fish, antibiotic exposure alters short‐chain fatty acid pools and host signalling in large yellow croaker ( *Larimichthys crocea* ), indicating that microbial metabolism can influence the availability of host‐accessible nutrients (Wang et al. 2025).

### 4.3 Conserved Functional Role of the Gut Microbiota in Salmonids

Whether this pattern is specific to juvenile chum salmon or general among salmonids is a fair question. Genome‐resolved metagenomics of Atlantic salmon ( *Salmo salar* ) shows that dominant gut lineages, particularly *Mycoplasma*, carry metabolic features consistent with nutritional interaction with the host, including potential contributions to amino acid and vitamin metabolism (Rasmussen et al. 2021, 2023). In rainbow trout ( *Oncorhynchus mykiss* ), shotgun metagenomics found functional partitioning of nutritional processes along the gut (Betiku et al. 2023). Combined metagenomic, transcriptomic and metabolomic analysis in Atlantic salmon demonstrated that gut microbiota generate and release metabolites, including vitamins, with putative benefit to the host (Vera‐Ponce de León et al. 2024). At the ecosystem level, thiamine in aquatic food webs is largely produced by bacteria and phytoplankton and transferred upward through trophic levels (Hylander et al. 2024). Our comparison adds to this by linking microbial biosynthetic capacity explicitly to modules that are incomplete in the host genome during early development.

Life stage is an important qualifier here. Adult and juvenile salmonid microbiomes are substantially different. In wild Atlantic salmon, gut community composition varies with life‐history phase and geography rather than being fixed (Llewellyn et al. 2016), and in farmed Atlantic salmon a recent genome catalogue resolved distinct presmolt and postsmolt communities (Kale et al. 2025). Stage‐specific microbial signatures have also been described across embryonic and early intestinal development (Lokesh et al. 2019). Our data therefore describe one developmental window and should not be extrapolated to returning adults, and conversely the *Mycoplasma*‐dominated picture from adult and postsmolt salmon should not be assumed to hold at 35 days posthatching.

### 4.4 Key Microbial Contributors and Their Interpretation

The modules compensating for essential amino acid and vitamin biosynthesis were largely attributed to sequences affiliated with *Acinetobacter*, *Bartonella*‐like organisms and *Enterococcus*, within Proteobacteria and Firmicutes. This contrasts with Atlantic salmon and rainbow trout, where *Mycoplasma* is often numerically dominant, especially in older individuals (Dehler et al. 2017; Lyons et al. 2017; Rasmussen et al. 2021). A *Mycoplasma*‐affiliated lineage was present in our dataset as well, particularly in the untreated library, so the difference is one of dominance rather than presence. The functional theme is nonetheless consistent: whichever lineages dominate, amino acid biosynthesis modules are carried by the community rather than by the host, which points to functional convergence across different bacterial taxa depending on host age, environment and diet.

*A. calcoaceticus* ‐like sequences contributed to thiamine and riboflavin modules (M00127, M00125) and to leucine and valine/isoleucine biosynthesis (M00432, M00019). *Enterococcus* spp. contributed to leucine and threonine modules, which adds a possible amino acid supply role to this genus's established use as a probiotic in aquaculture (Gatesoupe 1999; Luan et al. 2023).

Bartonellaceae are classically arthropod‐vectored symbionts and pathogens of mammals (Breitschwerdt and Kordick 2000). There is no established literature describing an ecological role for *Bartonella* in fish or in freshwater gut environments; the few aquatic‐associated records concern marine mammals rather than teleosts. We therefore cannot point to a documented ecological function for these organisms in a salmon gut, and we do not wish to invent one. Three explanations remain open. The sequences may derive from a genuinely novel, uncharacterized member of the Bartonellaceae or a sister lineage adapted to the fish gut. They may derive from environmental bacteria introduced with tank water or feed and transiting the gut rather than colonizing it. Or, most conservatively, they may reflect homology‐based assignment of conserved biosynthetic genes to the nearest sequenced relative, which for many alphaproteobacterial amino acid pathways is a *Bartonella* genome. Distinguishing these possibilities requires genome binning with average nucleotide identity assessment or targeted isolation, neither of which the present dataset supports.

The biotin result requires the same caution in stronger form. The dominant assignments were to intracellular endosymbionts of arthropods and protists, organisms that plainly do not inhabit a salmon gut. The reference genome database used by Genomaple has limited representation of fish‐associated bacteria, and functionally similar biotin biosynthesis genes are assigned to the closest available reference genome even when that genome belongs to a distant organism. Many endosymbionts have small, streamlined genomes in which biotin biosynthesis is unusually well conserved, which makes them disproportionately likely to appear as top matches for biotin pathway genes from any source. We therefore report the biotin module as present in the community while declining to assign it to a specific taxon.

### 4.5 Implications for Hatchery Management and Conservation

Chum salmon have not been reared through a complete life cycle in captivity, and Japanese fisheries depend on hatchery‐raised juveniles released to supplement natural recruitment at a scale that has fallen along with adult returns over recent decades (Kitada and Kishino 2025). The physiological quality of juveniles before ocean entry is therefore directly relevant to management. Linking specific microbial functions to biosynthetic pathways during early development offers a framework for reconsidering hatchery diets from a microbiome perspective. Diet is a realistic lever since feed composition is one of the strongest determinants of salmonid gut community structure and its functional profile (Gajardo et al. 2017; Wang et al. 2021). Formulated diets designed to support particular microbial taxa, or targeted use of probiotics and prebiotics, could plausibly improve nutritional condition before release, but this remains a hypothesis requiring controlled trials.

Antibiotic use in salmonid rearing is relevant in the same context and cuts the other way. Antimicrobial treatment reshapes the gut community and the metabolic pathways it encodes. In farmed Atlantic salmon, successive florfenicol‐medicated feeding altered gut community composition, intestinal inflammatory response and growth pattern in otherwise healthy fish. In European seabass, antibiotic treatment shifted the metabolic potential of the gut community, including pathways for amino acid metabolism and cofactor and vitamin metabolism, and recovery was incomplete and gut‐region dependent (Rosado et al. 2020). If the biosynthetic modules we describe do contribute to the juvenile nutrient pool, then antibiotic exposure during the hatchery phase would be expected to remove part of that contribution at precisely the developmental point where demand is highest. This has not been tested in chum salmon and is a concrete, tractable experiment: compare module completion and growth in antibiotic‐treated and untreated fry on an identical diet.

### 4.6 Limitations and Future Directions

Several limitations should be stated. Gut contents were small, so microbial DNA from 12 individuals was pooled into treatment‐level metagenomes; the analysis therefore does not capture interindividual variation. Because the libraries were pooled and unreplicated, no inferential statistics were applied and all comparisons among treatments are descriptive. The assignment of functional genes to taxa depends on the coverage of reference genome databases, and species‐level names here indicate best matches rather than validated identifications. Genome binning followed by average nucleotide identity assessment and classification against the Genome Taxonomy Database would place these assignments on much firmer ground (Chaumeil et al. 2020; Parks et al. 2022), and is the obvious next methodological step once host depletion improves further. A complete KEGG module indicates biosynthetic potential rather than realized flux.

Two experimental approaches would move this from potential to quantity. Metatranscriptomics would establish which of these modules are actually transcribed in the juvenile gut, and paired with metabolomics would connect transcription to metabolite pools. Stable isotope tracing addresses the harder question of source attribution. In practice, an isotopically labelled substrate is supplied to the system, for example a 13C‐ or 15N‐labelled precursor that only the microbial pathway can use, and the labelled atoms are then followed into downstream metabolites and into host tissue. If host protein or host tissue pools acquire label that can only have passed through a microbial biosynthetic route, the microbial contribution has been demonstrated rather than inferred, and the enrichment ratio gives its approximate magnitude. Applied to nucleic acids, the same principle identifies which community members assimilate the substrate. This approach has recently been used to map microbiome‐to‐host‐organ metabolite transfer in mammals (Xiao et al. 2024), and adapting it to hatchery fry, where diet and water can be controlled completely, is feasible.

### 4.7 Concluding Remarks

We developed a workflow that makes gene‐centric shotgun metagenomics practical for the very small guts of juvenile chum salmon, and used it to compare the metabolic capacity of the host genome and its gut microbiota at 35 days posthatching. The comparison shows that several biosynthetic pathways for essential amino acids and B‐vitamins are incomplete in the host genome but encoded with high completeness in the gut microbiome. These observations offer a practical framework for microbiome‐aware strategies to enhance the quality and resilience of hatchery‐reared juveniles, and they point to the importance of understanding microbial nutritional roles in wild salmon populations facing a changing ocean.

## Author Contributions

**Subrata Kumar Ghosh:** conceptualization, investigation, methodology, validation, formal analysis, visualization, writing – original draft, writing – review and editing, data curation. **Susumu Hyodo:** methodology, resources, validation, writing – review and editing. **Mai A. Wassel:** methodology, formal analysis, visualization, writing – review and editing, data curation. **Koji Hamasaki:** conceptualization, methodology, supervision, project administration, validation, funding acquisition, writing – review and editing, software. **Marty Kwok‐Shing Wong:** methodology, validation, writing – review and editing, resources.

## Funding

This work was supported by the University of Tokyo and the Ministry of Education, Culture, Sports, Science and Technology.

## Ethics Statement

This study was performed according to the Guidelines for the Care and Use of Animals approved by the Animal Experiment Committee of The University of Tokyo and complied with the ARRIVE guidelines.

## Conflicts of Interest

The authors declare no conflicts of interest.

## Supporting information


**Table S1:** Sequencing and DNA summary statistics for the four metagenomic libraries.
**Table S2:** Species‐level composition of the gut microbiome in each library (relative abundance, %), based on ribosomal protein assignments.
**Table S3:** KEGG module completion ratios for the chum salmon genome (117,599 protein‐coding sequences, 100% ribosome completeness).
**Table S4:** KEGG module completion ratios for the 35 DPH original DNA library (no pretreatment).
**Table S5:** KEGG module completion ratios for the 35 DPH NEBNext + 17‐cycle PCR library.
**Table S6:** KEGG module completion ratios for the 35 DPH NEBNext + whole‐genome amplification library.

## Data Availability

Raw data are deposited in FigShare (https://doi.org/10.6084/m9.figshare.31316443) and included in the Supporting Information. Raw sequences are deposited in the DDBJ repository and are publicly available under the BioProject: PRJDB42818 (PSUB049008) and Run: DRR1079436‐DRR1079439 (subrata_bisei‐0006_Run_0001–0004).

## References

[emi70448-bib-0001] American Veterinary Medical Association . 2007. AVMA Guidelines on Euthanasia. American Veterinary Medical Association.

[emi70448-bib-0002] Arai, W. , T. Taniguchi , S. Goto , et al. 2018. “MAPLE 2.3.0: An Improved System for Evaluating the Functionomes of Genomes and Metagenomes.” Bioscience, Biotechnology, and Biochemistry 82: 1515–1517. 10.1080/09168451.2018.1476122. 29792119

[emi70448-bib-0003] Betiku, O. C. , C. J. Yeoman , T. G. Gaylord , S. L. Ishaq , G. C. Duff , and W. M. Sealey . 2023. “Evidence of a Divided Nutritive Function in Rainbow Trout ( *Oncorhynchus mykiss* ) Midgut and Hindgut Microbiomes by Whole Shotgun Metagenomic Approach.” Aquacult Rep 30: 101601. 10.1016/j.aqrep.2023.101601.

[emi70448-bib-0004] Boutin, S. , C. Sauvage , L. Bernatchez , C. Audet , and N. Derome . 2014. “Inter‐Individual Variations of the Fish Skin Microbiota: Host Genetics Basis of Mutualism?” PLoS One 9: e102649. 10.1371/journal.pone.0102649. PMC4113282 25068850

[emi70448-bib-0005] Breitschwerdt, E. B. , and D. L. Kordick . 2000. “ *Bartonella* Infection in Animals: Carriership, Reservoir Potential, Pathogenicity, and Zoonotic Potential for Human Infection.” Clinical Microbiology Reviews 13: 428–438. 10.1128/CMR.13.3.428. PMC88941 10885985

[emi70448-bib-0006] Chaumeil, P.‐A. , A. J. Mussig , P. Hugenholtz , and D. H. Parks . 2020. “GTDB‐Tk: A Toolkit to Classify Genomes With the Genome Taxonomy Database.” Bioinformatics 36: 1925–1927. 10.1093/bioinformatics/btz848. PMC7703759 31730192

[emi70448-bib-0007] Dehler, C. E. , C. J. Secombes , and S. A. Martin . 2017. “Seawater Transfer Alters the Intestinal Microbiota Profiles of Atlantic Salmon ( *Salmo salar* L.).” Scientific Reports 7: 13877. 10.1038/s41598-017-13249-8. PMC5654775 29066818

[emi70448-bib-0008] Douglas, G. M. , V. J. Maffei , J. R. Zaneveld , et al. 2020. “PICRUSt2 for Prediction of Metagenome Functions.” Nature Biotechnology 38: 685–688. 10.1038/s41587-020-0548-6. PMC7365738 32483366

[emi70448-bib-0009] Egerton, S. , S. Culloty , J. Whooley , C. Stanton , and R. P. Ross . 2018. “The Gut Microbiota of Marine Fish.” Frontiers in Microbiology 9: 873. 10.3389/fmicb.2018.00873. PMC5946678 29780377

[emi70448-bib-0010] Feng, Q. , W. D. Chen , and Y. D. Wang . 2018. “Gut Microbiota: An Integral Moderator in Health and Disease.” Frontiers in Microbiology 9: 151. 10.3389/fmicb.2018.00151. PMC5826318 29515527

[emi70448-bib-0011] Gajardo, K. , A. Jaramillo‐Torres , T. M. Kortner , et al. 2017. “Alternative Protein Sources in the Diet Modulate Microbiota and Functionality in the Distal Intestine of Atlantic Salmon ( *Salmo salar* ).” Applied and Environmental Microbiology 83: e02615‐16. 10.1128/AEM.02615-16. PMC5311410 27986728

[emi70448-bib-0013] Gatesoupe, F. J. 1999. “The Use of Probiotics in Aquaculture.” Aquaculture 180: 147–165. 10.1016/S0044-8486(99)00187-8.

[emi70448-bib-0015] Ghosh, S. K. , M. K. S. Wong , S. Hyodo , and K. Hamasaki . 2025. “Shifting Seas and First Feeds: Gut Microbiome Dynamics in Juvenile Chum Salmon ( *Oncorhynchus keta* ) and Their Climate Vulnerability.” Current Research in Microbial Sciences 9: 100452. 10.1016/j.crmicr.2025.100452. PMC12355085 40822128

[emi70448-bib-0017] Hylander, S. , H. Farnelid , E. Fridolfsson , et al. 2024. “Thiamin (Vitamin B1, Thiamine) Transfer in the Aquatic Food Web From Lower to Higher Trophic Levels.” PLoS One 19: e0308844. 10.1371/journal.pone.0308844. PMC11611157 39621630

[emi70448-bib-0018] Jain, C. , L. M. Rodriguez‐R , A. M. Phillippy , K. T. Konstantinidis , and S. Aluru . 2018. “High Throughput ANI Analysis of 90K Prokaryotic Genomes Reveals Clear Species Boundaries.” Nature Communications 9: 5114. 10.1038/s41467-018-07641-9. PMC6269478 30504855

[emi70448-bib-0019] Kale, V. , G. Baldi , M. Beracochea , et al. 2025. “A Bacterial and Viral Genome Catalogue From Atlantic Salmon Highlights Diverse Gut Microbiome Compositions at Pre‐ and Post‐Smolt Life Stages.” Animal Microbiome 7: 85. 10.1186/s42523-025-00453-5. PMC12341145 40790604

[emi70448-bib-0021] Kitada, S. , and H. Kishino . 2025. “Hatcheries to High Seas: Climate Change Connections to Salmon Marine Survival.” Ecology and Evolution 15: e71504. 10.1002/ece3.71504. PMC12122101 40454215

[emi70448-bib-0022] Konstantinidis, K. T. , and J. M. Tiedje . 2005. “Genomic Insights That Advance the Species Definition for Prokaryotes.” Proceedings of the National Academy of Sciences of the United States of America 102: 2567–2572. 10.1073/pnas.0409727102. PMC549018 15701695

[emi70448-bib-0023] Lasken, R. S. 2012. “Genomic Sequencing of Uncultured Microorganisms From Single Cells.” Nature Reviews. Microbiology 10: 631–640. 10.1038/nrmicro2857. 22890147

[emi70448-bib-0024] Liu, H. , X. Guo , R. Gooneratne , et al. 2016. “The Gut Microbiome and Degradation Enzyme Activity of Wild Freshwater Fishes Influenced by Their Trophic Levels.” Scientific Reports 6: 24340. 10.1038/srep24340. PMC4829839 27072196

[emi70448-bib-0025] Llewellyn, M. S. , S. Boutin , S. H. Hoseinifar , and N. Derome . 2014. “Teleost Microbiomes: The State of the Art in Their Characterization, Manipulation and Importance in Aquaculture and Fisheries.” Frontiers in Microbiology 5: 207. 10.3389/fmicb.2014.00207. PMC4040438 24917852

[emi70448-bib-0026] Llewellyn, M. S. , P. McGinnity , M. Dionne , et al. 2016. “The Biogeography of the Atlantic Salmon ( *Salmo salar* ) Gut Microbiome.” ISME Journal 10: 1280–1284. 10.1038/ismej.2015.189. PMC5029221 26517698

[emi70448-bib-0027] Lokesh, J. , V. Kiron , D. Sipkema , J. M. O. Fernandes , and T. Moum . 2019. “Succession of Embryonic and the Intestinal Bacterial Communities of Atlantic Salmon ( *Salmo salar* ) Reveals Stage‐Specific Microbial Signatures.” MicrobiologyOpen 8: e00672. 10.1002/mbo3.672. PMC6460355 29897674

[emi70448-bib-0028] Luan, Y. , M. Li , W. Zhou , et al. 2023. “The Fish Microbiota: Research Progress and Potential Applications.” Engineering 29: 137–146. 10.1016/j.eng.2022.12.011.

[emi70448-bib-0029] Lyons, P. P. , J. F. Turnbull , K. A. Dawson , and M. Crumlish . 2017. “Phylogenetic and Functional Characterization of the Distal Intestinal Microbiome of Rainbow Trout *Oncorhynchus mykiss* From Both Farm and Aquarium Settings.” Journal of Applied Microbiology 122: 347–363. 10.1111/jam.13347. 27860093

[emi70448-bib-0030] Marotz, C. A. , J. G. Sanders , C. Zuniga , L. S. Zaramela , R. Knight , and K. Zengler . 2018. “Improving Saliva Shotgun Metagenomics by Chemical Host DNA Depletion.” Microbiome 6: 42. 10.1186/s40168-018-0426-3. PMC5827986 29482639

[emi70448-bib-0031] McCormick, S. D. 2013. “Smolt Physiology and Endocrinology.” In Fish Physiology, edited by E. Fishes , S. D. McCormick , A. P. Farrell , and C. J. Brauner , vol. 32, 199–251. Academic Press. 10.1016/B978-0-12-396951-4.00005-0.

[emi70448-bib-0032] Morita, H. , T. Kuwahara , K. Ohshima , et al. 2007. “An Improved DNA Isolation Method for Metagenomic Analysis of the Microbial Flora of the Human Intestine.” Microbes and Environments 22: 214–222. 10.1264/jsme2.22.214.

[emi70448-bib-0033] Nayak, S. K. 2010. “Role of Gastrointestinal Microbiota in Fish.” Aquaculture Research 41: 1553–1573. 10.1111/j.1365-2109.2010.02546.x.

[emi70448-bib-0034] Okubo, T. , A. Toyoda , K. Fukuhara , et al. 2021. “The Physiological Potential of Anammox Bacteria as Revealed by Their Core Genome Structure.” DNA Research 28: dsaa028. 10.1093/dnares/dsaa028. PMC7814187 33367889

[emi70448-bib-0035] Olsvik, P. A. , G. I. Hemre , and R. Waagbø . 2013. “Exploring Early Micronutrient Deficiencies in Rainbow Trout ( *Oncorhynchus mykiss* ) by Next‐Generation Sequencing Technology – From Black Box to Functional Genomics.” PLoS One 8: e69461. 10.1371/journal.pone.0069461. PMC3722103 23894486

[emi70448-bib-0036] Parks, D. H. , M. Chuvochina , C. Rinke , A. J. Mussig , P.‐A. Chaumeil , and P. Hugenholtz . 2022. “GTDB: An Ongoing Census of Bacterial and Archaeal Diversity Through a Phylogenetically Consistent, Rank Normalized and Complete Genome‐Based Taxonomy.” Nucleic Acids Research 50: D785–D794. 10.1093/nar/gkab776. PMC8728215 34520557

[emi70448-bib-0037] Pereira‐Marques, J. , A. Hout , R. M. Ferreira , et al. 2019. “Impact of Host DNA and Sequencing Depth on the Taxonomic Resolution of Whole Metagenome Sequencing for Microbiome Analysis.” Frontiers in Microbiology 10: 1277. 10.3389/fmicb.2019.01277. PMC6581681 31244801

[emi70448-bib-0038] Rasmussen, J. A. , P. Kiilerich , A. S. Madhun , et al. 2023. “Co‐Diversification of an Intestinal *Mycoplasma* and Its Salmonid Host.” ISME Journal 17: 682–692. 10.1038/s41396-023-01379-z. PMC10119124 36807409

[emi70448-bib-0039] Rasmussen, J. A. , K. R. Villumsen , D. A. Duchêne , et al. 2021. “Genome‐Resolved Metagenomics Suggests a Mutualistic Relationship Between *Mycoplasma* and Salmonid Hosts.” Communications Biology 4: 579. 10.1038/s42003-021-02105-1. PMC8121932 33990699

[emi70448-bib-0040] Rawls, J. F. , B. S. Samuel , and J. I. Gordon . 2004. “Gnotobiotic Zebrafish Reveal Evolutionarily Conserved Responses to the Gut Microbiota.” Proceedings of the National Academy of Sciences of the United States of America 101: 4596–4601. 10.1073/pnas.0400706101. PMC384792 15070763

[emi70448-bib-0041] Rombout, J. H. W. M. , L. Abelli , S. Picchietti , G. Scapigliati , and V. Kiron . 2011. “Teleost Intestinal Immunology.” Fish & Shellfish Immunology 31: 616–626. 10.1016/j.fsi.2010.09.001. 20832474

[emi70448-bib-0042] Rombout, J. H. W. M. , A. J. Taverne‐Thiele , and M. I. Villena . 1993. “The Gut‐Associated Lymphoid Tissue (GALT) of Carp ( *Cyprinus carpio* L.): An Immunocytochemical Analysis.” Developmental and Comparative Immunology 17: 55–66. 10.1016/0145-305X(93)90015-I. 8449251

[emi70448-bib-0043] Rosado, D. , R. Xavier , R. Severino , F. Tavares , J. Cable , and M. Pérez‐Losada . 2020. “Antibiotic Effect and Microbiome Persistence Vary Along the European Seabass Gut.” Scientific Reports 10: 9896. 10.1038/s41598-020-66622-5. PMC7305304 32561815

[emi70448-bib-0044] Semova, I. , J. D. Carten , J. Stombaugh , et al. 2012. “Microbiota Regulate Intestinal Absorption and Metabolism of Fatty Acids in the Zebrafish.” Cell Host & Microbe 12: 277–288. 10.1016/j.chom.2012.08.003. PMC3517662 22980325

[emi70448-bib-0045] Shi, Y. , G. Wang , H. C. H. Lau , and J. Yu . 2022. “Metagenomic Sequencing for Microbial DNA in Human Samples: Emerging Technological Advances.” International Journal of Molecular Sciences 23: 2181. 10.3390/ijms23042181. PMC8877284 35216302

[emi70448-bib-0046] Sullam, K. E. , S. D. Essinger , C. A. Lozupone , et al. 2012. “Environmental and Ecological Factors That Shape the Gut Bacterial Communities of Fish: A Meta‐Analysis.” Molecular Ecology 21: 3363–3378. 10.1111/j.1365-294X.2012.05552.x. PMC3882143 22486918

[emi70448-bib-0047] Takami, H. , T. Taniguchi , W. Arai , K. Takemoto , Y. Moriya , and S. Goto . 2016. “An Automated System for Evaluation of the Potential Functionome: MAPLE Version 2.1.0.” DNA Research 23: 467–475. 10.1093/dnares/dsw030. PMC5066172 27374611

[emi70448-bib-0048] Takami, H. , T. Taniguchi , Y. Moriya , T. Kuwahara , M. Kanehisa , and S. Goto . 2012. “Evaluation Method for the Potential Functionome Harbored in the Genome and Metagenome.” BMC Genomics 13: 699. 10.1186/1471-2164-13-699. PMC3541978 23234305

[emi70448-bib-0049] Vera‐Ponce de León, A. , T. Hensen , M. Hoetzinger , et al. 2024. “Genomic and Functional Characterization of the Atlantic Salmon Gut Microbiome in Relation to Nutrition and Health.” Nature Microbiology 9: 3059–3074. 10.1038/s41564-024-01830-7. 39402236

[emi70448-bib-0050] Wang, J. , A. Jaramillo‐Torres , Y. Li , et al. 2021. “Microbiota in Intestinal Digesta of Atlantic Salmon ( *Salmo salar* ), Observed From Late Freshwater Stage Until One Year in Seawater, and Effects of Functional Ingredients: A Case Study From a Commercial Sized Research Site in the Arctic Region.” Animal Microbiome 3: 14. 10.1186/s42523-021-00075-7. PMC7841887 33509296

[emi70448-bib-0051] Wang, J. , M. Lei , Y. Liu , Z. Wang , J. Fan , and Y. Wang . 2025. “Insights From Antibiotic‐Induced Gut Microbiota Dysbiosis and Metabolic Changes in the Gut and Hypothalamus Into the Microbiota–Gut–Brain Axis in Large Yellow Croaker ( *Larimichthys crocea* ).” Aquaculture 599: 742157. 10.1016/j.aquaculture.2025.742157.

[emi70448-bib-0052] Wemheuer, F. , J. A. Taylor , R. Daniel , et al. 2020. “Tax4Fun2: Prediction of Habitat‐Specific Functional Profiles and Functional Redundancy Based on 16S rRNA Gene Sequences.” Environmental Microbiomes 15: 11. 10.1186/s40793-020-00358-7. PMC8067651 33902725

[emi70448-bib-0053] Xiao, X. , Y. Zhou , X. Li , et al. 2024. “13C‐Stable Isotope Resolved Metabolomics Uncovers Dynamic Biochemical Landscape of Gut Microbiome–Host Organ Communications in Mice.” Microbiome 12: 96. 10.1186/s40168-024-01808-x. PMC11094917 38750595

